# Acupuncture is associated with lower accidental injury risk among patients with Parkinson’s disease: a nationwide retrospective cohort study

**DOI:** 10.3389/fneur.2026.1889885

**Published:** 2026-09-30

**Authors:** Hui-Jer Chou, Ming-Jen Wang, Shun-Ku Lin

**Affiliations:** 1 Department of Chinese Medicine, Taipei City Hospital, Taipei, Taiwan; 2 Department of Physical Therapy and Assistive Technology, National Yang Ming Chiao Tung University, Taipei, Taiwan; 3 Institute of Traditional Medicine, School of Medicine, National Yang Ming Chiao Tung University, Taipei, Taiwan; 4 Institute of Public Health, School of Medicine, National Yang Ming Chiao Tung University, Taipei, Taiwan; 5 General Education Center, University of Taipei, Taipei, Taiwan

**Keywords:** accidental injury, acupuncture, falls, Nationwide cohort, Parkinson’s disease, public health, real-world evidence, Taiwan

## Abstract

**Background:**

Patients with Parkinson’s disease (PD) have a high risk of accidental injuries because motor and non-motor symptoms can impair gait, balance, cognition, and autonomic function. Acupuncture has been reported to improve gait and balance, but its association with long-term injury prevention in PD remains unclear.

**Methods:**

We conducted a nationwide retrospective cohort study using Taiwan’s National Health Insurance Research Database. Patients newly diagnosed with PD between 2001 and 2012 were followed through 2018. Propensity score matching (PSM) was used to improve comparability between patients who received acupuncture and those who did not. The PSM cohort was designated as the primary analysis, and a multivariable analysis of the full cohort was supportive. Cox proportional hazards models estimated adjusted hazard ratios (aHRs) and 95% confidence intervals (CIs).

**Results:**

Among 24,467 patients with PD, 7,830 (32.0%) received acupuncture. Accidental injuries occurred in 15.2% of patients in the acupuncture group and 17.1% of patients in the non-acupuncture group. In the primary PSM analysis, acupuncture was associated with a lower risk of hospitalization for accidental injury (aHR, 0.67; 95% CI, 0.42–0.85). The association was strongest for accidental falls, although no simple dose–response relationship was observed across acupuncture-session categories. The supportive full-cohort analysis yielded a similar direction of association (aHR, 0.60; 95% CI, 0.39–0.76).

**Conclusion:**

In this nationwide claims-based cohort, acupuncture use was associated with a lower risk of hospitalization for accidental injury among patients newly diagnosed with PD. Because residual confounding and differences in care-seeking may remain, prospective studies with clinical measures of PD severity are warranted.

## Graphical abstract

**Figure:**
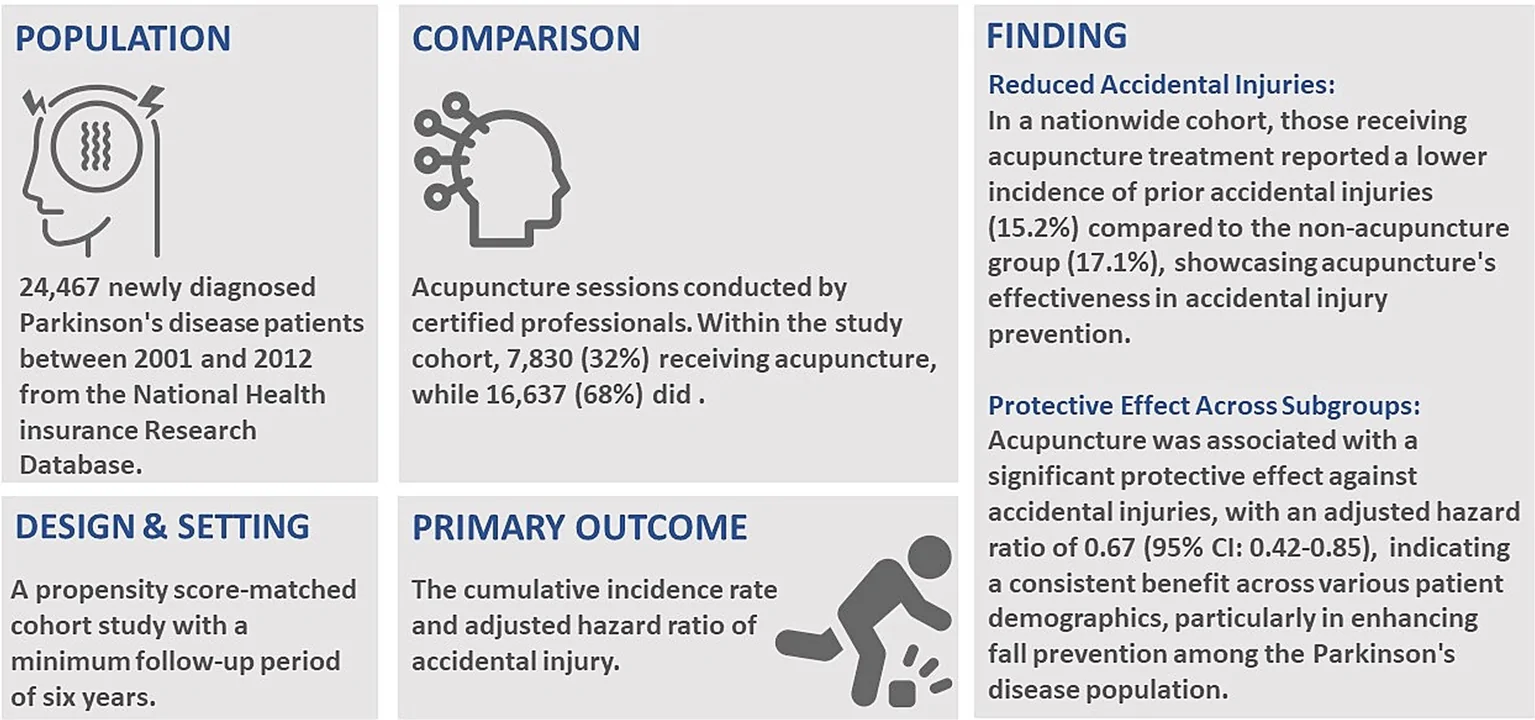

## 1 Introduction

Patients with Parkinson’s disease (PD) are at heightened risk of accidental injury because the disease affects mobility, balance, autonomic function, cognition, and visual–spatial processing. Motor features such as tremor, rigidity, bradykinesia, and postural instability increase fall risk (1, 2), while non-motor symptoms, including orthostatic hypotension and cognitive impairment, may further compromise safety in daily life (2, 3). Accidental injuries can lead to hospitalization, pain, functional decline, reduced quality of life, and increased dependence (4).

Acupuncture is used as a complementary therapy for PD. Previous randomized studies have suggested that acupuncture may improve gait and balance parameters, including stride length, cadence, gait speed, and measures related to walking and balance (5, 6). These findings raise the possibility that acupuncture could influence clinically meaningful safety outcomes in PD. However, whether acupuncture is associated with a lower long-term risk of accidental injury in real-world PD populations has not been well established.

The primary objective of this study was to evaluate the association between acupuncture treatment and the risk of accidental injury among patients with PD. We also estimated injury incidence and examined whether the association varied across demographic and clinical subgroups.

## 2 Materials and methods

### 2.1 Study design

We conducted a retrospective cohort study of patients diagnosed with PD and classified them according to whether they received acupuncture after diagnosis. Participants were followed for at least 6 years, where available, to assess accidental injury incidence. Propensity score matching was applied to improve between-group comparability and reduce confounding by indication. The study was approved by the Research Ethics Committee of Taipei City Hospital (TCHIRB-11103028-E), which waived the requirement for individual informed consent because only de-identified administrative claims data were analyzed.

### 2.2 Setting

This study encompassed Taiwan and its affiliated islands (Penghu, Kinmen, Matsu, and Lanyu), using National Health Insurance (NHI) data from 2000 to 2018. The data were derived from the 2000 Longitudinal Generation Tracking Database (LGTD 2000), which was constructed by randomly selecting 2 million individuals from Taiwan’s population of approximately 23 million in 2000 and following them longitudinally. The database is representative of the Taiwanese population and provides comprehensive long-term inpatient and outpatient claims data. More than 99% of the Taiwanese population is enrolled in the NHI, and claims are reviewed by the National Health Insurance Administration to support data accuracy and completeness. Taiwanese administrative claims have also supported prior population-based investigations of traditional Chinese medicine use and health outcomes (7–9).

### 2.3 Participants

We included individuals newly diagnosed with Parkinson’s disease by a neurologist from 2001 through 2012, identified using ICD-9-CM code 332 or ICD-10-CM codes G20 and G21. To improve diagnostic specificity, eligible patients were required to have at least three outpatient visits or at least one hospitalization for Parkinson’s disease and to have used antiparkinsonian medication continuously for at least 1 month.

Conversely, this study implemented specific exclusion criteria. Excluded were individuals diagnosed during the preliminary screening period (2000–2001), ensuring the inclusion of only those with new diagnoses within the study’s designated period. Additionally, patients with a history of accidental injuries or illnesses predating the screening period or their initial Parkinson’s disease diagnosis were omitted. The study also excluded individuals with concurrent diagnoses of other mental disorders, such as bipolar disorder or schizophrenia; only those with an exclusive diagnosis of Parkinson’s disease were considered. Participants who had not received any pharmacological treatment for Parkinson’s disease and those with incomplete or absent health records were likewise excluded from the study.

Each participant was followed from cohort entry until the earliest of hospitalization for accidental injury, death, withdrawal from Taiwan’s National Health Insurance for more than 1 year, or December 31, 2018. The primary PSM model included age at PD diagnosis, sex, insured amount, urbanization level, and the baseline comorbidities reported in Table 1. Rheumatoid arthritis and dementia were additionally included as covariates in the Cox models because of their potential associations with mobility, frailty, cognition, and injury risk.

**Table 1:** Demographic characteristics and comorbidities of the Parkinson’s disease cohort.

Variables	Original cohort			Propensity score-matched cohort		
	Acupuncture	Non-acupuncture	Standardized mean difference	Acupuncture	Non-acupuncture	Standardized mean difference
	No. (%)	No. (%)		No. (%)	No. (%)	
Total	7,830 (100.0%)	16,637 (100.0%)	—	5,375	5,375	
Age at diagnosis			−0.295*			−0.0001
<60	690 (8.8%)	714 (4.3%)		468 (8.7%)	473 (8.8%)	
60–70	1,044 (13.3%)	1,398 (8.4%)		722 (13.4%)	708 (13.2%)	
70–80	1,134 (14.5%)	1,452 (8.7%)		787 (14.6%)	772 (14.4%)	
≥ 80	4,962 (63.4%)	13,073 (78.6%)		3,398 (63.2%)	3,423 (63.7%)	
Sex			−0.160			<0.0001
Male	4,344 (55.5%)	7,902 (47.5%)		2,984 (55.5%)	3,014 (56.1%)	
Female	3,486 (44.5%)	8,735 (52.5%)		2,392 (44.5%)	2,362 (43.9%)	
Urbanization			−0.192			0.0002
Very high	2,430 (31.0%)	3,732 (22.4%)		1,650 (30.7%)	1,683 (31.3%)	
High	4,482 (57.2%)	10,403 (62.5%)		3,044 (56.6%)	3,044 (56.6%)	
Moderate	660 (8.4%)	1,638 (9.8%)		452 (8.4%)	456 (8.5%)	
Low	258 (3.3%)	864 (5.2%)		230 (4.3%)	192 (3.6%)	
Insured amount			0.121			<0.0001
Dependent	2,862 (36.6%)	6,390 (38.4%)		1,948 (36.2%)	1,987 (37.0%)	
1–19,999	2,478 (31.6%)	5,934 (35.7%)		1,716 (31.9%)	1,682 (31.3%)	
20,000–39,999	1,998 (25.5%)	3,786 (22.8%)		1,371 (25.5%)	1,357 (25.2%)	
≥ 40,000	492 (6.3%)	527 (3.2%)		341 (6.4%)	350 (6.5%)	
Comorbidity						
Diabetes mellitus	3,324 (42.5%)	7,074 (42.5%)	−0.0013	2,285 (42.5%)	2,262 (42.1%)	0.0027
Cerebrovascular accident	4,596 (58.7%)	10,584 (63.6%)	−0.1010	3,156 (58.7%)	3,156 (58.7%)	<0.0001
Chronic kidney disease	1,494 (19.1%)	3,126 (18.8%)	0.0075	1,027 (19.1%)	1,037 (19.3%)	−0.0015
Coronary heart disease	1,374 (17.5%)	2,662 (16.0%)	0.0930	941 (17.5%)	931 (17.3%)	0.0016
Hypertension	5,592 (71.4%)	12,341 (74.2%)	−0.0621	3,877 (72.1%)	3,800 (70.7%)	0.0171
Hyperlipidemia	3,786 (48.4%)	5,832 (35.1%)	0.2722*	2,628 (48.9%)	2,628 (48.9%)	<0.0001
Osteoarthritis	3,012 (38.5%)	4,386 (26.4%)	0.2608*	2,090 (38.9%)	2,090 (38.9%)	<0.0001

PSM covariates are displayed with baseline distributions. Rheumatoid arthritis and dementia were additional Cox-model covariates; they were not used to define matched pairs.

*Absolute standardized mean difference greater than 0.20, indicating meaningful between-group imbalance before matching.

### 2.4 Acupuncture

This study included acupuncture and related procedures covered by Taiwan’s National Health Insurance, including manual acupuncture, electroacupuncture, cupping, bloodletting, scraping (Gua Sha), thermotherapy, moxibustion, and special acupuncture for the eyes. The LGTD 2000 records treatment date, location, physician, and frequency. Treatments provided outside the licensed medical system, acupressure, and exercise-based guidance were not captured.

### 2.5 Main outcome

The primary outcome was hospitalization for accidental injury after PD diagnosis. Accidental injuries included falls, poisoning, drowning, burns, traffic accidents, and other externally caused injuries. Injury categories were identified using the ICD-9-CM Supplementary Classification of External Causes of Injury and Poisoning (E000-E999) and ICD-10-CM External Causes of Morbidity (V00-Y99), recorded in the EXT_CODE1 and EXT_CODE2 fields. The date of hospitalization for accidental injury was used to calculate follow-up time.

### 2.6 Variables

Comorbidities considered as potential confounders included diabetes mellitus (DM), cerebrovascular accident (CVA), chronic kidney disease (CKD), coronary heart disease (CHD), hypertension (HTN), rheumatoid arthritis (RA), hyperlipidemia (HL), osteoarthritis (OA), and dementia (DEM). These conditions may worsen balance, mobility, cognition, or general frailty and thereby increase the risk of falls and other accidental injuries (2, 4, 10, 11).

Demographic factors such as patients’ sex, age at the time of medical consultation, family income, and place of residence may influence the prognosis of PD and were included as confounders in the analysis (12). Because PD is a typical neurodegenerative disease and most patients are diagnosed at age 65 years or older, patients were categorized by age at PD diagnosis into four groups: young onset (<60 years), early elderly (60–70 years), middle elderly (70–80 years), and advanced elderly (≥ 80 years). The age and sex distribution in the LGTD 2000 for the year 2000 did not differ significantly from the entire population of Taiwan, supporting the representativeness of this sample (13). Insurance location in the LGTD 2000 was associated with residential area and was classified into four urbanization levels: very high, high, medium, and low. Because NHI premiums are based on income, participants were categorized into four income brackets: dependent with no income, low income (monthly 1–19,999 TWD), middle income (monthly 20,000–39,999 TWD), and high income (monthly >40,000 TWD).

Sedative-hypnotic medications, including benzodiazepines (BZDs), barbiturates, and Z-drugs, were included because they may increase accidental injury risk through sedation, dizziness, impaired coordination, and cognitive effects (11, 14). These medication effects may be particularly important in patients with PD, who already have impaired motor control and postural stability.

### 2.7 Bias

Confounding by indication was a major concern because patients who seek acupuncture may differ from nonusers in PD severity, functional impairment, disease progression, social support, access to care, and health-care engagement. PSM was used to improve comparability for measured baseline characteristics, and multivariable Cox models further adjusted for demographic factors, comorbidities, and sedative-hypnotic medication use. Nevertheless, claims data do not contain direct measures such as Unified Parkinson’s Disease Rating Scale scores, gait or balance assessments, or functional status; therefore, residual confounding by disease severity and a healthy-user or care-engagement effect may remain. We also addressed immortal time bias by excluding the interval between cohort entry and the first acupuncture treatment from acupuncture-group follow-up time.

### 2.8 Statistical methods

The descriptive analysis section of this study included counts and percentages for each variable in both the acupuncture and control groups. The standardized mean difference was calculated to compare the differences in comorbidities and demographic variables between the groups. Survival curves were plotted using the Kaplan–Meier method, and the log-rank test was employed to estimate whether the difference in the risk of accidental injuries in PD patients treated with acupuncture was statistically significant. Additionally, this study calculated the incidence rate of accidental injuries per 1,000 person-years in different PD populations.

Cox proportional hazards models were used to estimate adjusted hazard ratios (aHRs) and 95% confidence intervals (CIs) after controlling for age, sex, insured amount, urbanization level, comorbidities, and sedative-hypnotic medication use. The analysis in the PSM cohort was prespecified as the primary analysis because it provided the most comparable treatment groups. The multivariable Cox analysis in the full cohort was treated as supportive. Additional analyses examined consistency across demographic and clinical subgroups and associations according to acupuncture-session frequency, follow-up duration, injury type, and Charlson Comorbidity Index (CCI) category.

Subgroup analyses were conducted to explore the consistency and potential variability of acupuncture’s protective effects across different patient profiles and treatment characteristics. This approach enabled a more nuanced understanding of how the effectiveness of acupuncture might vary in specific patient groups or under different clinical scenarios.

Statistical significance was defined as a two-sided alpha level of 0.05. A standardized mean difference greater than 0.2 was considered evidence of meaningful between-group imbalance. All analyses were performed using SAS, version 9.4.

## 3 Results

### 3.1 Descriptive data

From the two million individuals in the LGTD 2000, we identified 24,467 patients newly diagnosed with PD who met the study criteria and were at risk of accidental injury. Of these, 7,830 (32.0%) received acupuncture and 16,637 (68.0%) did not. During follow-up, accidental injury occurred in 1,190 patients (15.2%) in the acupuncture group and 2,844 patients (17.1%) in the non-acupuncture group (Figure 1).

**Figure 1:**
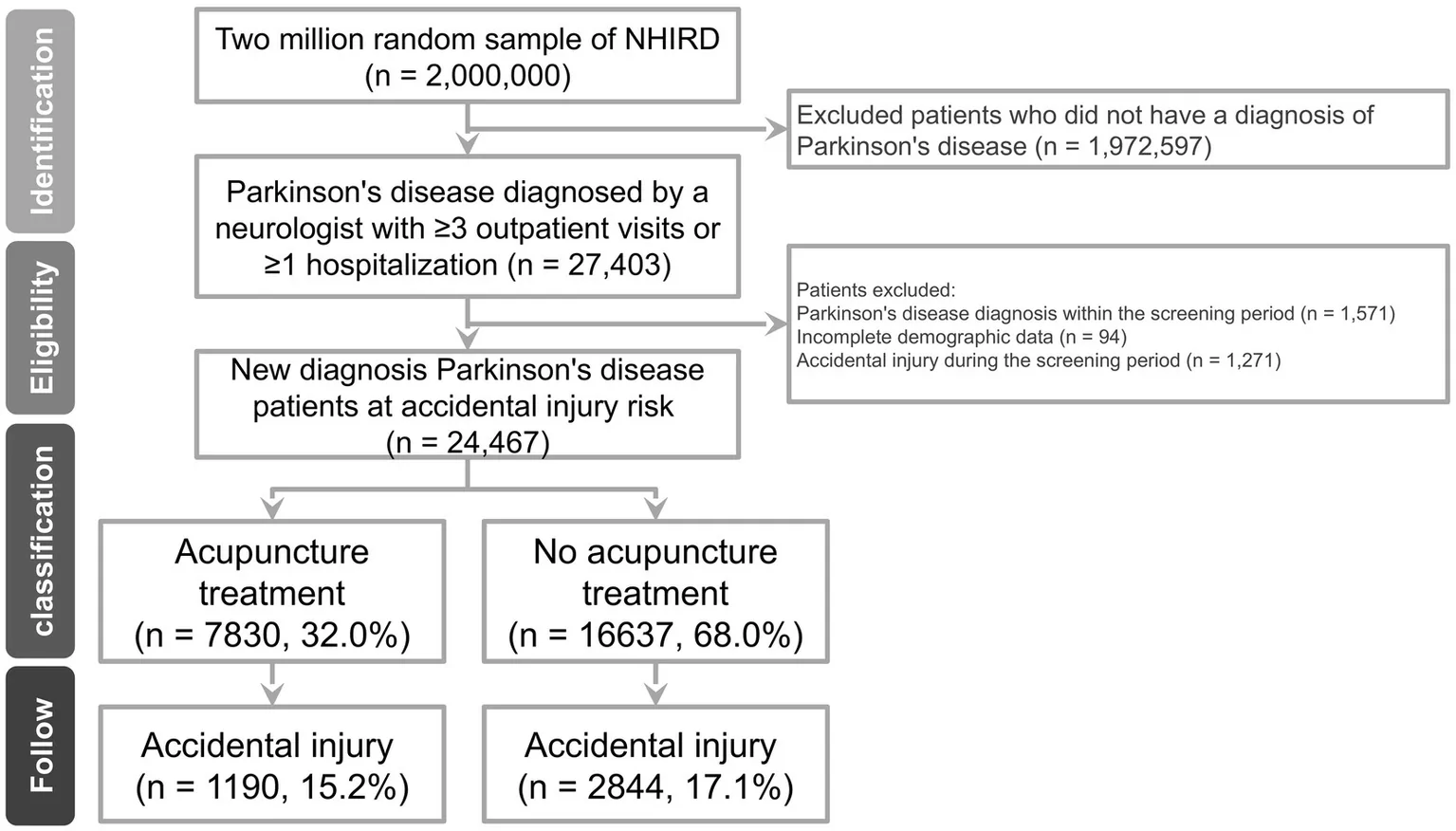
Flowchart depicting participant selection and hospitalization for accidental injury among patients with Parkinson’s disease. Eligible patients had a neurologist-recorded diagnosis with at least three outpatient visits or at least one hospitalization.

Baseline demographic and clinical characteristics differed between the acupuncture and non-acupuncture groups (Table 1). Patients receiving acupuncture were less likely to be aged 80 years or older and more likely to be male, live in very highly urbanized areas, and have higher insured income. Several comorbidities also differed between groups. Propensity score matching was therefore used in subsequent analyses to balance measured covariates.

Before matching, the acupuncture group had a lower prevalence of cerebrovascular accident than the non-acupuncture group, but higher prevalences of rheumatoid arthritis, hyperlipidemia, and osteoarthritis. These imbalances supported the use of propensity score matching before estimating the association between acupuncture and accidental injury risk.

### 3.2 Outcome data

Kaplan–Meier analysis showed a lower cumulative incidence of accidental injury in the acupuncture group over the 18-year observation period (Figure 2). Accidental injuries occurred in 15.2% of patients receiving acupuncture and 17.1% of those not receiving acupuncture. Mean event-free survival time was 17.2 years (standard error, 0.131) in the acupuncture group and 16.2 years (standard error, 0.116) in the non-acupuncture group. The difference between groups was statistically significant by log-rank test (*p* < 0.001).

**Figure 2:**
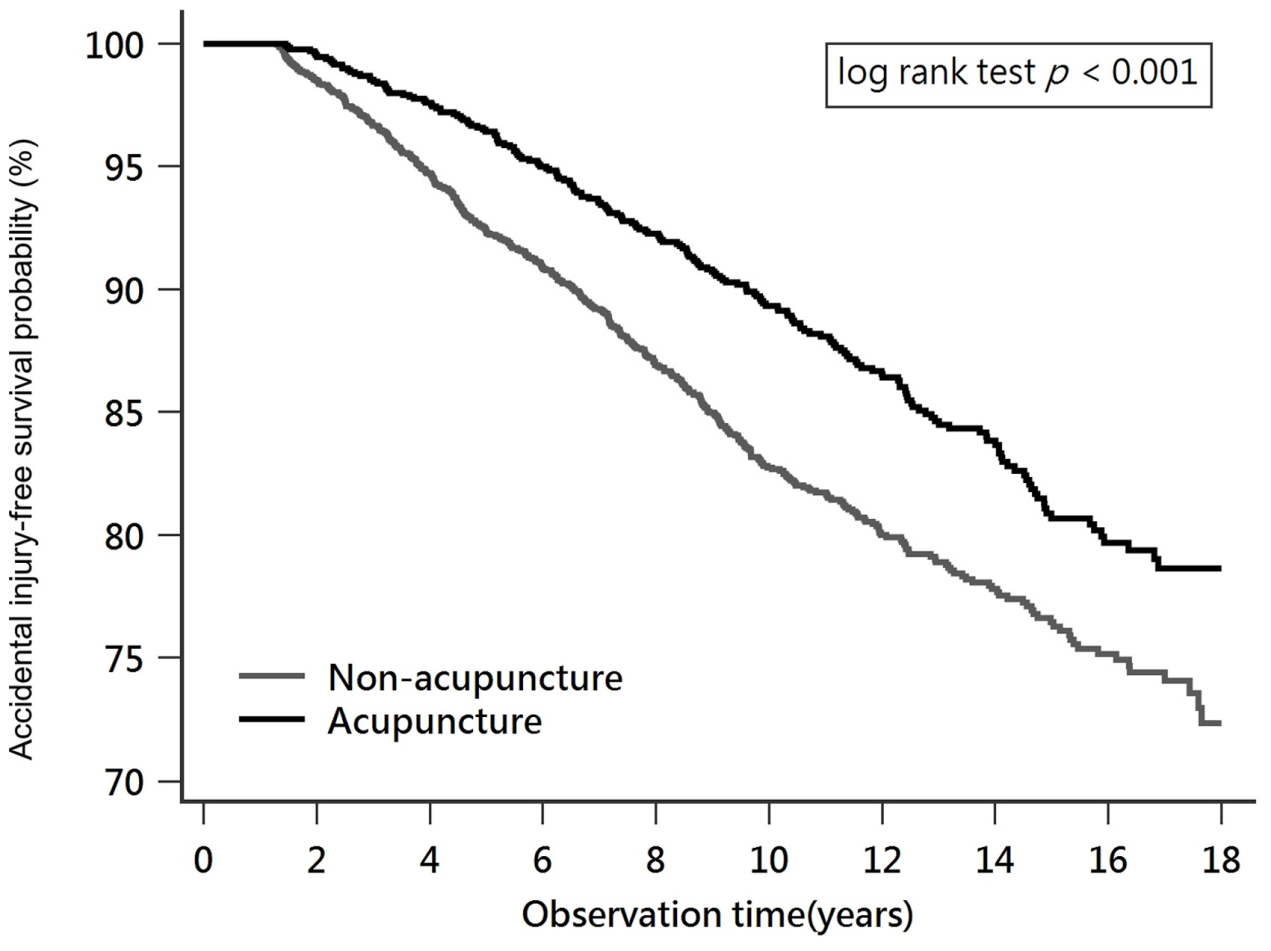
Kaplan–Meier analysis of accidental injury-free survival among patients with Parkinson’s disease according to acupuncture treatment. Accidental injuries occurred in 15.2% of patients receiving acupuncture and 17.1% of patients not receiving acupuncture.

### 3.3 Primary and supportive analyses

In the primary PSM analysis, acupuncture was associated with a lower risk of hospitalization for accidental injury (aHR, 0.67; 95% CI, 0.42–0.85; Figure 3). Older age was strongly associated with higher risk, with the greatest hazard among patients aged 80 years or older. Cerebrovascular accident, chronic kidney disease, hyperlipidemia, dementia, osteoarthritis, and sedative-hypnotic medication use were also associated with higher injury hazards.

**Figure 3:**
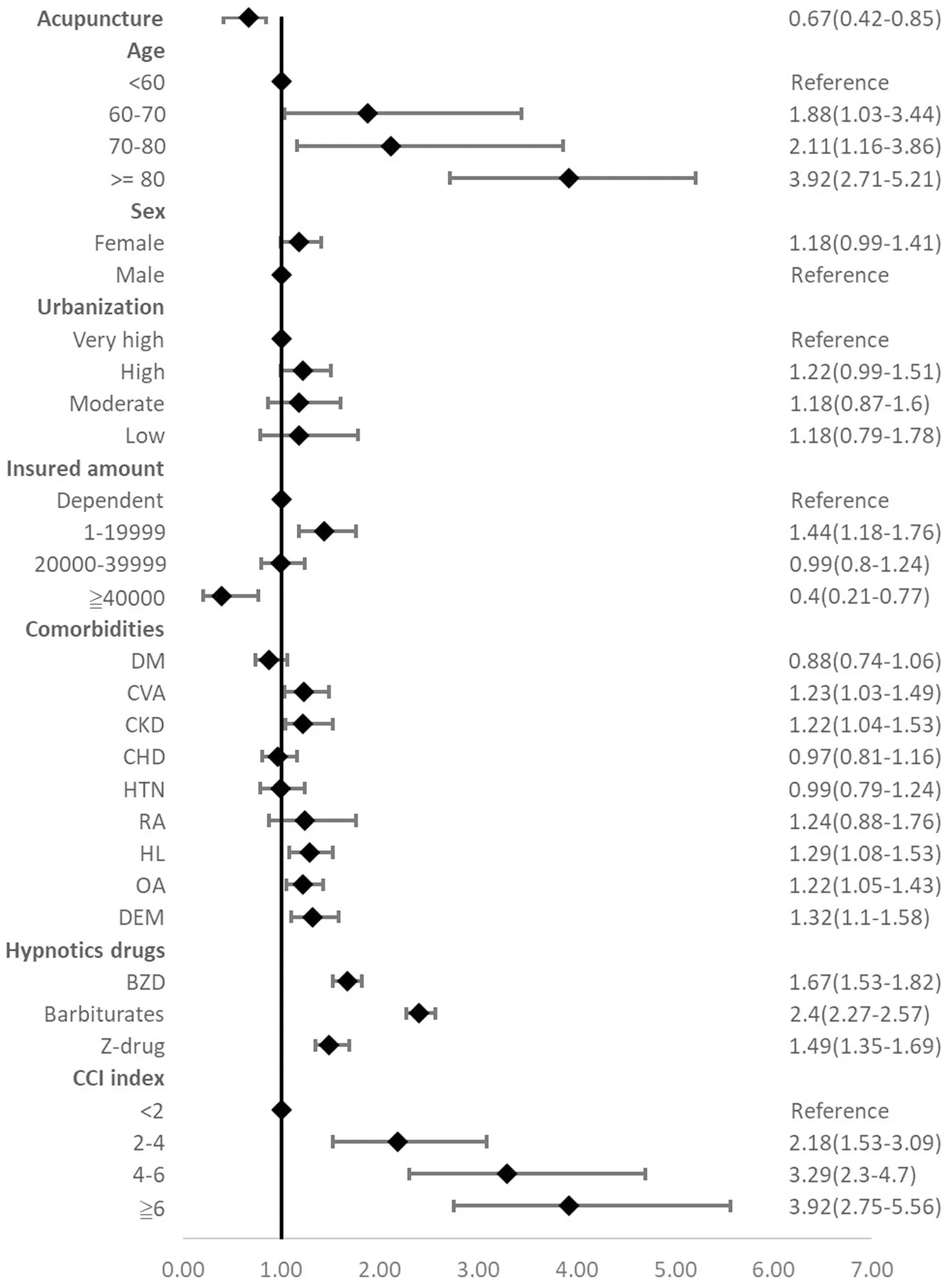
Adjusted hazard ratios for hospitalization for accidental injury in the propensity score-matched cohort of patients with Parkinson’s disease. DM, diabetes mellitus; CVA, cerebrovascular accident; CKD, chronic kidney disease; CHD, coronary heart disease; HTN, hypertension; RA, rheumatoid arthritis; HL, hyperlipidemia; OA, osteoarthritis; DEM, dementia; BZD, benzodiazepine; CCI, Charlson Comorbidity Index.

The supportive full-cohort analysis showed the same direction of association. Acupuncture users had an injury incidence rate of 12.5 per 1,000 person-years compared with 17.5 per 1,000 person-years among nonusers, with an adjusted hazard ratio of 0.60 (95% CI, 0.39–0.76; Table 2). This estimate is presented as supportive because baseline covariate imbalance was greater before matching.

**Table 2:** Risk of accidental injury in terms of acupuncture treatment and covariates among patients with Parkinson’s disease.

Variables	*N*	Person-years	Incidence rate per 1,000 person-years	Crude hazard ratio (95% confidence interval)	Adjusted hazard ratio (95% confidence interval)
Acupuncture					
Yes	1,190	94968.0	12.5	0.54 (0.35–0.68)	0.60 (0.39–0.76)
No	2,844	162093.1	17.5	Reference	Reference
Sex					
Female	2,440	132456.1	18.4	1.45 (1.22–1.72)	1.26 (1.11–1.54)
Male	1,594	124605.0	12.8	Reference	Reference
Age					
<60	150	35135.4	4.3	Reference	Reference
60–70	212	23466.7	9.0	2.29 (1.24–4.01)	2.03 (1.09–3.78)
70–80	505	44794.2	11.3	2.5 (1.52–4.46)	2.22 (1.33–4.25)
≥ 80	3,167	153670.2	20.6	4.87 (3.31–6.48)	4.51 (3.01–5.89)
Insurance amount					
Dependent	1,367	99024.2	13.8	Reference	Reference
1–19,999	1,638	79826.4	20.5	1.77 (1.43–2.21)	1.56 (1.31–1.97)
20,000–39,999	925	64924.0	14.2	1.19 (0.92–1.49)	1.05 (0.86–1.40)
≥ 40,000	104	13287.0	7.8	0.52 (0.25–0.94)	0.45 (0.22–0.86)
Urbanization					
Very high	887	73607.6	12.1	Reference	Reference
High	2,535	146428.2	17.3	1.42 (1.19–1.86)	1.34 (1.1–1.74)
Moderate	399	25508.3	15.6	1.35 (0.99–1.88)	1.27 (0.91–1.79)
Low	213	11517.6	18.5	1.48 (1.02–2.15)	1.31 (0.91–1.96)
Comorbidity					
Diabetes mellitus	1,736	107447.7	16.2	1.14 (0.91–1.28)	1.01 (0.79–1.2)
Cerebrovascular accident	2,408	152717.8	15.8	1.06 (0.85–1.47)	1.02 (0.87–1.13)
Chronic kidney disease	1,012	47510.1	21.3	1.44 (1.16–1.92)	1.32 (1.04–1.71)
Coronary heart disease	710	42121.8	16.9	1.16 (1.02–1.40)	1.03 (0.92–1.28)
Hypertension	3,249	182773.8	17.8	1.15 (0.90–1.49)	1.05 (0.83–1.35)
Hyperlipidemia	2,305	109651.1	21.0	1.52 (1.26–1.80)	1.39 (1.17–1.64)
Osteoarthritis	2,017	81120.0	24.8	1.51 (1.23–1.74)	1.34 (1.27–1.55)
Dementia	2,094	89970.2	23.3	1.60 (1.37–1.95)	1.41 (1.19–1.72)
Sedative hypnotic drugs					
Benzodiazepines	2,281	84919.0	26.9	2.17 (1.87–2.52)	1.89 (1.71–2.00)
Barbiturates	659	23290.5	28.3	2.85 (2.67–3.05)	2.66 (2.50–2.83)
Z-drug	2,149	90839.9	23.7	1.89 (1.74–2.01)	1.67 (1.53–1.91)

Subgroup analyses suggested that the association between acupuncture and lower accidental injury risk was generally consistent, although the magnitude and statistical significance varied by patient characteristics (Figure 4). The association was not statistically significant among patients younger than 60 years, those living in areas with low urbanization, those with the highest insurance coverage, and those using barbiturates.

**Figure 4:**
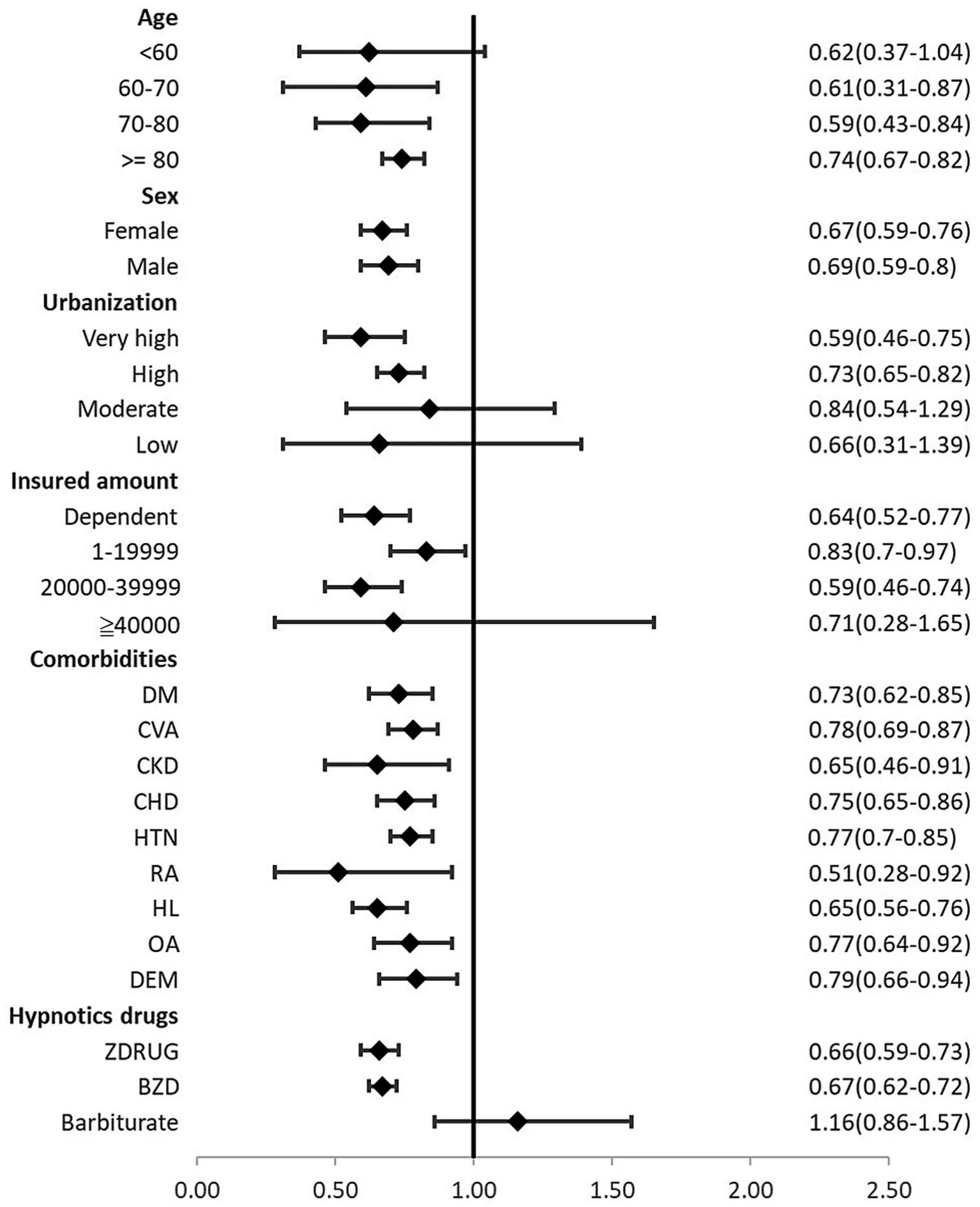
Adjusted hazard ratios for factors influencing the association between acupuncture and accidental injury risk in patients with Parkinson’s disease. DM, diabetes mellitus; CVA, cerebrovascular accident; CKD, chronic kidney disease; CHD, coronary heart disease; HTN, hypertension; RA, rheumatoid arthritis; HL, hyperlipidemia; OA, osteoarthritis; DEM, dementia; BZD, benzodiazepine.

### 3.4 Other analyses

Analyses of acupuncture frequency did not show a simple dose–response relationship (Figure 5). Fewer than six sessions were not associated with a statistically significant reduction in injury risk (aHR, 0.86; 95% CI, 0.55–1.36). The lowest aHRs were observed among patients receiving 12–18 sessions (aHR, 0.63; 95% CI, 0.44–0.90) and 18–24 sessions (aHR, 0.49; 95% CI, 0.20–0.83), while the association was attenuated among those receiving more than 24 sessions (aHR, 0.73; 95% CI, 0.51–0.98). This non-monotonic pattern does not establish a treatment dose–response effect. Frequent users may have had greater disease severity, progressive motor impairment, persistent symptoms, or stronger engagement with health services, any of which could influence both treatment frequency and hospitalization risk.

**Figure 5:**
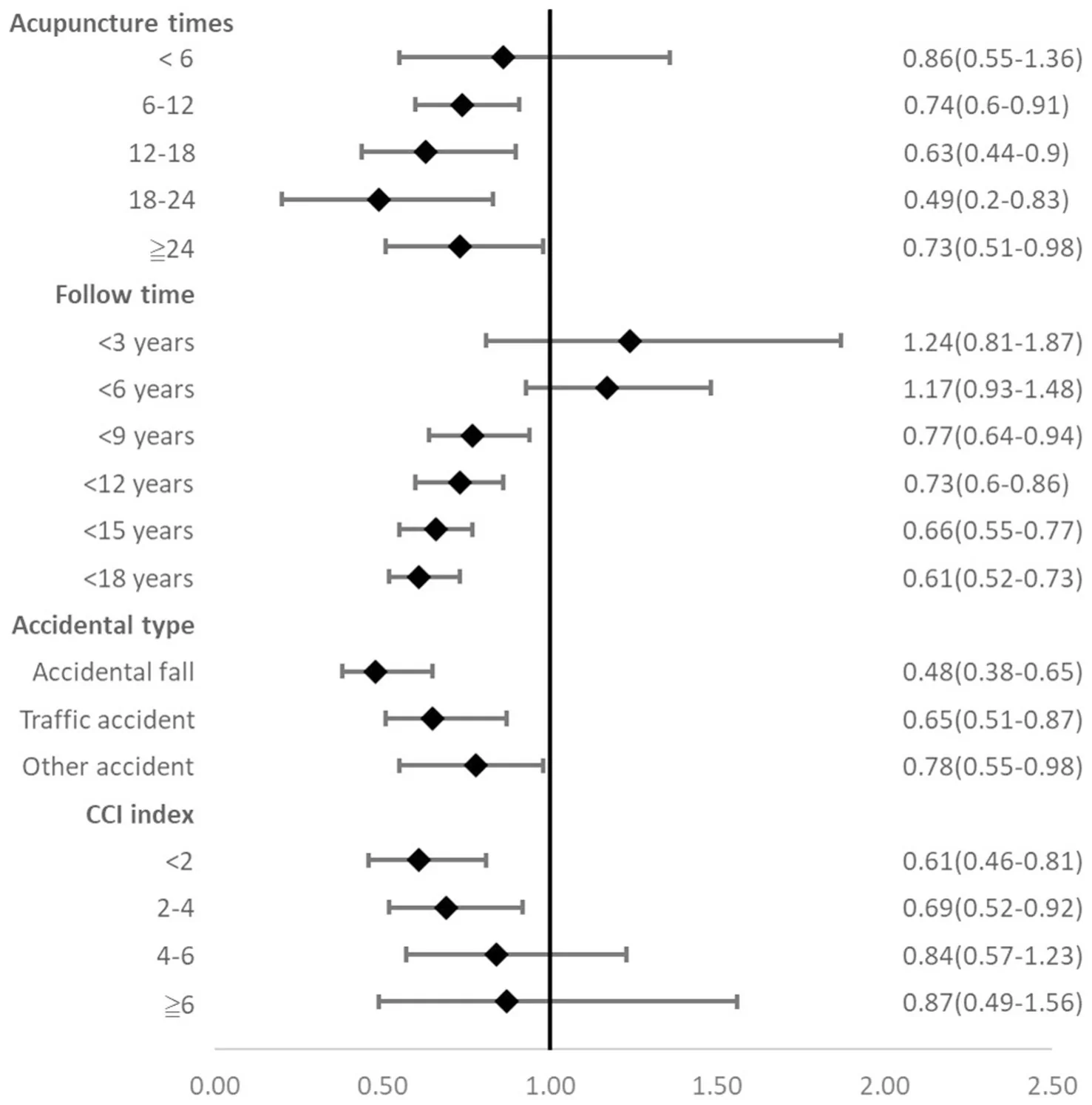
Adjusted hazard ratios for hospitalization for accidental injury according to acupuncture-session frequency, follow-up duration, injury type, and Charlson Comorbidity Index score.

By injury type, acupuncture showed the strongest association with reduced risk of accidental falls (aHR, 0.48; 95% CI, 0.38–0.65).

The protective association appeared weaker among patients with greater comorbidity burden. Patients with Charlson Comorbidity Index scores of 4–6 had an aHR of 0.84 (95% CI, 0.57–1.23), and those with scores of 6 or higher had an aHR of 0.87 (95% CI, 0.49–1.56).

## 4 Discussion

### 4.1 Key results

In this nationwide cohort of 24,467 patients newly diagnosed with PD, the primary PSM analysis found that acupuncture use was associated with a lower risk of hospitalization for accidental injury (aHR, 0.67; 95% CI, 0.42–0.85). The association was broadly consistent across most patient categories and appeared strongest for accidental falls. However, the observational design cannot establish that acupuncture caused the lower risk. The non-monotonic frequency pattern and attenuation among patients receiving more than 24 sessions are compatible with residual confounding by disease progression, motor impairment, care-seeking behavior, and treatment indication.

The absence of a simple dose–response relationship suggests that session frequency alone may not represent treatment intensity, indication, disease severity, or clinical context. Frequent acupuncture use may identify patients with greater underlying clinical need, whereas acupuncture users as a group may also differ from nonusers in preventive behavior, social support, health literacy, or continuity of care. These competing mechanisms could bias the observed association in either direction. The present results should therefore be interpreted as associations that warrant testing in prospective studies with detailed clinical measures.

These findings are consistent with previous Taiwanese claims-based research showing that Chinese herbal medicine use was associated with lower fracture risk among patients with PD (12), and extend that literature by focusing on acupuncture and a broader accidental injury outcome. A recent meta-analysis also reported beneficial effects of acupuncture on motor function in patients with Parkinson’s disease (15).

### 4.2 Generalizability

This study included only patients newly diagnosed with PD during the study period and excluded those with accidental injury before cohort entry. This design strengthened temporal clarity for the first observed injury event but did not allow evaluation of recurrent injuries or secondary parkinsonism. Because acupuncture is reimbursed and culturally familiar in Taiwan, effect estimates may differ in health systems with other access, reimbursement, or treatment patterns. Nevertheless, the study is relevant to neurologists, rehabilitation clinicians, and acupuncture practitioners worldwide because it highlights injury hospitalization as a patient-important outcome and underscores the need to integrate acupuncture, when used, with established fall-risk assessment and multidisciplinary PD care rather than view it as a substitute for standard management.

### 4.3 Limitations

Several limitations should be considered. First, the retrospective cohort design depends on the accuracy and completeness of administrative claims data. Second, the LGTD 2000 does not include detailed clinical measures such as Unified Parkinson’s Disease Rating Scale scores, gait or balance assessments, imaging findings, disease duration before diagnosis, or functional status. Residual confounding by PD severity, progression, frailty, preventive behavior, social support, and health-care engagement may therefore remain despite PSM and multivariable adjustment. Third, the outcome definition required hospitalization for accidental injury. Injuries managed only in outpatient clinics or emergency departments without admission were not captured, and the decision to hospitalize may differ by clinical severity, access, social support, or care-seeking behavior. Differential hospitalization could therefore influence the observed association. Fourth, detailed acupuncture information, including specific acupoints, stimulation techniques, treatment rationale, and practitioner-level variation, was unavailable. The findings estimate an overall association for NHI-reimbursed acupuncture-related care and should not be interpreted as establishing the efficacy of a particular acupuncture protocol or a causal injury-prevention effect.

## 5 Conclusion

In this nationwide retrospective cohort study, acupuncture use was associated with a lower risk of hospitalization for accidental injury among patients newly diagnosed with PD, with the strongest association observed for accidental falls. The findings are hypothesis-generating and should be interpreted in light of residual confounding and potential differences in care engagement and hospitalization. Prospective studies incorporating PD severity, gait, balance, functional status, and treatment details are needed before acupuncture can be considered an injury-prevention strategy.

## Data Availability

The data analyzed in this study is subject to the following licenses/restrictions: The datasets analyzed in this study are subject to restrictions imposed by Taiwan’s National Health Insurance Research Database and the Health and Welfare Data Science Center, Ministry of Health and Welfare, Taiwan. The data are de-identified administrative health claims data and are not publicly available. Access is limited to qualified researchers who submit an official application and obtain approval from the Health and Welfare Data Science Center. Requests to access the dataset should be directed to the Health and Welfare Data Science Center (HWDC), Ministry of Health and Welfare, Taiwan. Information on data application procedures is available at: https://dep.mohw.gov.tw/DOS/np-2497-113.html.
